# Supplementary material for: Photocatalytic CO2 reduction using La-Ni bimetallic sites within a covalent organic framework
Source: Nat Commun. 2023 Apr 29;14:2473. doi: 10.1038/s41467-023-37545-2 (PMC10148855; doi:10.1038/s41467-023-37545-2)
Supplement: Supplementary file 1 — Supplementary Information [file 41467_2023_37545_MOESM1_ESM.pdf]

Supplementary Information

Photocatalytic CO_2 reduction using La-Ni bimetallic sites within a covalent organic framework

[1] State Key Laboratory of Silicate Materials for Architectures, School of Materials Science and Engineering, Wuhan University of Technology, Wuhan 430070, P. R. China.

[2] College of Science, Huazhong Agricultural University, Wuhan 430070, P. R. China.

[3] Faculty of Materials Science and Chemistry, China University of Geosciences, Wuhan 430070, P. R. China.

[4] Univ. Grenoble-Alpes, CEA, CNRS, IRIG/SyMMES, STEP, 38000 Grenoble, France.

[5] Institut Européen des Membranes, IEM, UMR 5635, Université Montpellier, ENSCM, CNRS, Montpellier 34000, France.

[6] Shanghai Synchrotron Radiation Facility (SSRF), Shanghai Institute of Applied Physics, Chinese Academy of Sciences, Shanghai 200120, P. R. China.

[*] Corresponding authors:

Prof. Yueli Liu, E-mail: **lylliuwhut@whut.edu.cn**

Prof. Shengyao Wang, E-mail: **wangshengyao@mail.hzau.edu.cn**

Prof. Keqiang Chen, E-mail: **chenkeqiang@cug.edu.cn**

Prof. Peter Reiss, E-mail: **peter.reiss@cea.fr**

[†] These authors contributed equally: Min Zhou, Zhiqing Wang, Aohan Mei.

Supplementary Notes

Supplementary Note 1.

Compared with the N 1s characteristic peaks of Phen/COF-5 (398.9 eV, C=N), a slight upshift of N 1s peaks (399.9 eV) in LaNi-Phen/COF-5 is observed (Supplementary Fig. 8)[1]. The increase of the binding energy is attributed to the decrease of the electron cloud density of the Phen nitrogen, resulting from the electron transfer from nitrogen atoms in Phen to La or Ni ions. Besides, the N 1s signal of LaNi-Phen/COF-5 may be deconvoluted into two types of peaks located at 399.2 and 401.1 eV, corresponding to metal-N and C-N, respectively, which is caused by the coordination of nitrogen atoms with Ni and La ions[2].

Supplementary Note 2.

The coordination between N atoms in Phen and La/Ni ions is also confirmed by Fourier Transform Infrared (FTIR) spectroscopy (Supplementary Figs. 9a, 9b). The FTIR spectra of COF-5 colloid indicate the typical stretching bands originating from B-O, C-O, and B-C vibrations at around 1344, 1242, and 1020 cm^{-1}, respectively, revealing the existence of boronic ester groups with the characteristic five-membered ring[3]. These peaks are well-matched with the FTIR spectrum of LaNi-Phen/COF-5, indicating that the loading of LaNi-Phen has a negligible effect on the molecular structure of the COF-5 colloid. Furthermore, in the FTIR spectrum of LaNi-Phen/COF-5, the C=N stretching bonds at 1602 cm^{-1} and the C-H bands at 842 and 718 cm^{-1} reveal a slight bathochromic shift compared with those in the starting material of Phen (1643, 854 and 740 cm^{-1}, respectively), indicating that Ni-N and La-N bonds between the $LaCl_3·6H_2O$ and

$NiCl_2 \cdot 6H_2O$ starting compounds and the Phen groups are formed[4]. As expected, La-Phen/COF-5 and Ni-Phen/COF-5 show similar characteristics of the vibrational peaks.

Supplementary Note 3.

The permanent porosity of LaNi-Phen/COF-5 is investigated by N_2 adsorption isotherm measurements at 77 K (Supplementary Fig. 11), exhibiting the decrease in the Brunauer-Emmett-Teller (BET) surface area (1326 m^2g^{-1}) compared to that of pure COF-5 colloid (1490 m^2g^{-1}) due to the occupancy of the partial pore spaces after impregnation. As expected, the implantation of LaNi-Phen results in a decrease in porosity, which is consistent with the specific surface area shown in Supplementary Fig. 12. Although the specific surface area of LaNi-Phen/COF-5 is slightly lower than that of COF-5 colloid, it exhibits a much higher CO_2 adsorption capacity, which proves that the integration of LaNi-Phen into COF-5 colloid is conducive to improve the adsorption of CO_2 molecules (Supplementary Fig. 14).

Supplementary Note 4.

To further evaluate the stability of the as-prepared La/Ni diatomic photocatalysts, the heterogeneity experiments were performed by removing the catalyst from the reaction medium at t=4 h by centrifugation. The photocatalytic CO_2 reduction experiments were carried out with the filtrate (catalyst removed) under the same reaction conditions, which is a process of the gas-liquid phase. When the catalyst was removed, the generated gases, such as CO and H_2, were vented from the sealed system (Supplementary Fig. 27), indicating that the diatomic species of LaNi-Phen may be stably confined in the pores of COF-5 colloid without shedding during the

photocatalytic CO_2 reduction process.

Supplementary Note 5.

The carrier lifetime of the photogenerated charges is further evaluated by time-resolved photoluminescence (TRPL) measurements using an excitation wavelength of 365 nm, indicating charge transfer processes (Supplementary Fig. 34)[5]. The average carrier lifetime of the catalysts is summarized in Supplementary Table 6. Photoelectrochemical measurements are carried out to verify the charge carrier transfer rate of the catalysts. The response curve of LaNi-Phen/COF-5 shows the largest photocurrent density when the electrodes are illuminated with ultraviolet-visible light ($\lambda \geqslant 350$ nm), indicating the enhanced electrical conductivity and prolonged carrier lifetime of the photogenerated charges (Supplementary Fig. 36). The enhancement of the photocurrent intensity contributes to the improved efficiency for the photocatalytic CO_2 reduction. Electrochemical impedance spectroscopy (EIS) is applied to determine the electron transport characteristics of the catalysts. As shown in Supplementary Fig. 35, among all the photocatalysts, a remarkably decreased radius of the Nyquist plot is observed after loading of the COF-5 colloids with the combination of La/Ni metallic complexes, demonstrating the lower resistance for charge transfer separation in the case of LaNi-Phen/COF-5. To sum up, integrating La-Ni dual-atom sites into the COF-5 colloid leads to synergistic effects, which are the origin of the observed outstanding photoelectric performance of the LaNi-Phen/COF-5 catalyst.

Supplementary Figures

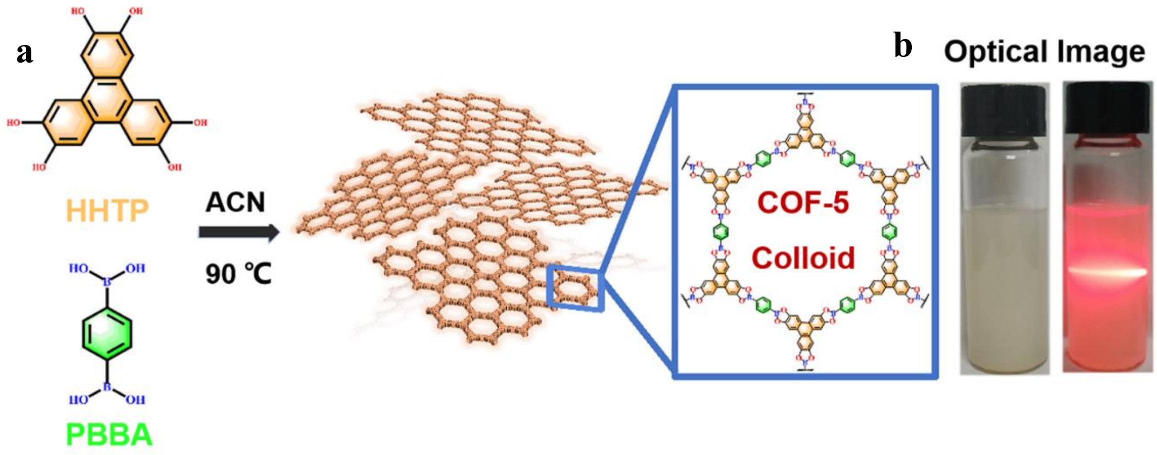

Supplementary Fig. 1 Synthesis diagram of boroxine-linked COF-5 colloid. a Synthesis diagram of boroxine-linked COF-5 colloid through solvothermal condensation of HHTP (5 mM) and PBBA. **b** Photograph of COF-5 colloid (left) and the corresponding Tyndall effect (right), indicating the particle size of the COF-5 colloids is within the nanoscale.

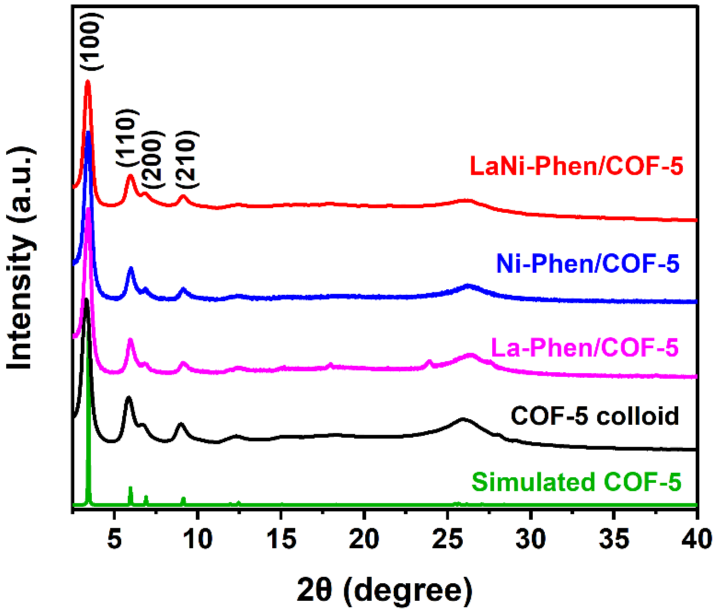

Supplementary Fig. 2 Powder XRD patterns. Powder XRD patterns of simulated COF-5, experimental COF-5 colloid, La-Phen/COF-5, Ni-Phen/COF-5 and LaNi-Phen/COF-5, demonstrating their very similar crystalline structures, indicating that the crystalline structure of the COF-5 colloid remains unchanged in LaNi-Phen/COF-5. The experimental patterns also match well the simulated one (the broad peak in the 25-30° range increases from the (001) reflection of COF-5)[3].

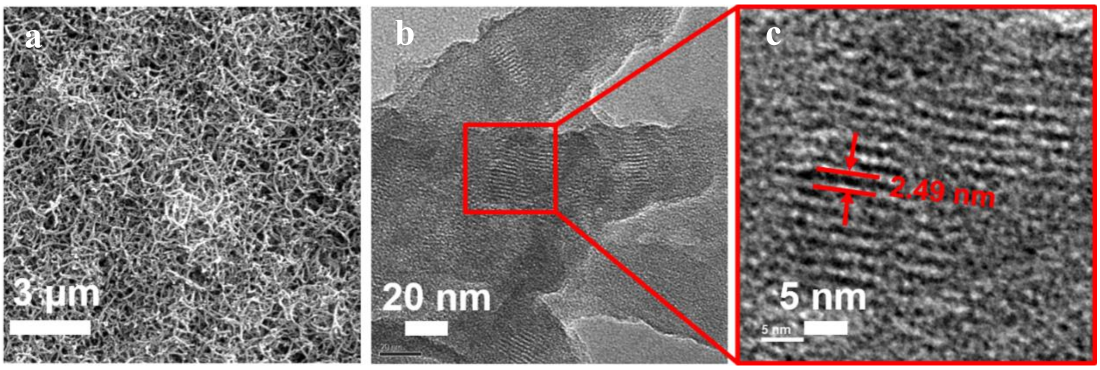

Supplementary Fig. 3 Morphology of COF-5 colloid. a SEM image. **b** TEM image. **c** HRTEM image of the enlarged area. Panel **a** shows its one-dimensional (1D) nanorods morphology with 70-80 nm in width. Panel **b** indicates the existence of the regular fringes corresponding to the pore size of 2.49 nm.

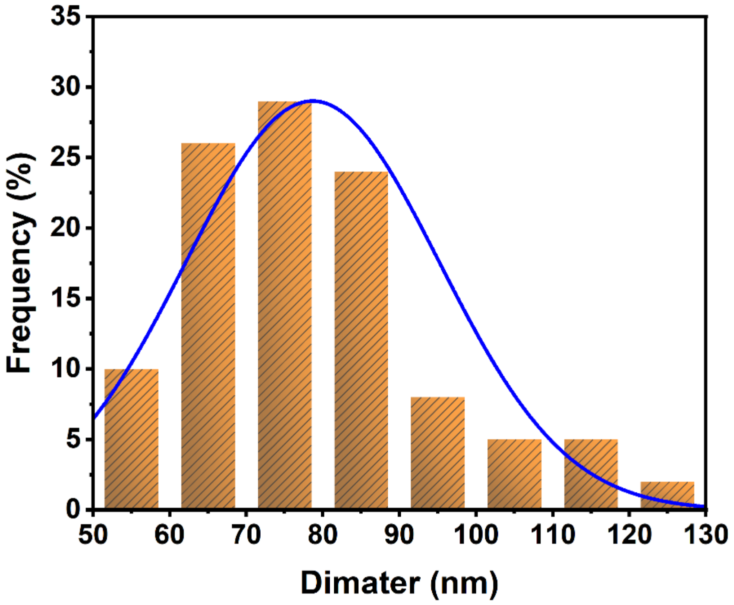

Supplementary Fig. 4 Particle width distribution. Particle width distribution of COF-5 colloid, showing the width of COF-5 colloid is mainly in the range of 70-80 nm.

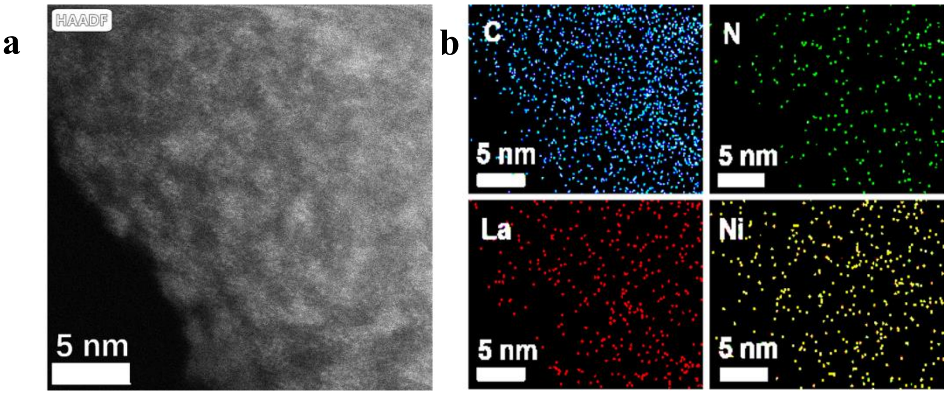

Supplementary Fig. 5 HAADF-STEM observation of LaNi-Phen/COF-5**. a** Atomic-resolution HAADF-STEM image. **b** energy-dispersive spectroscopy mapping images.

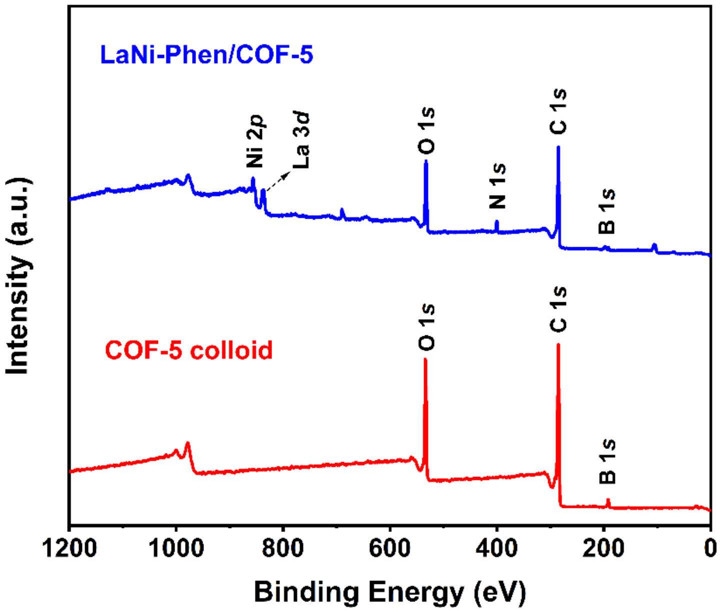

Supplementary Fig. 6 XPS full spectra. XPS spectra of COF-5 colloid and LaNi-Phen/COF-5, supporting the existence of C, N, O, La and Ni in the catalysts.

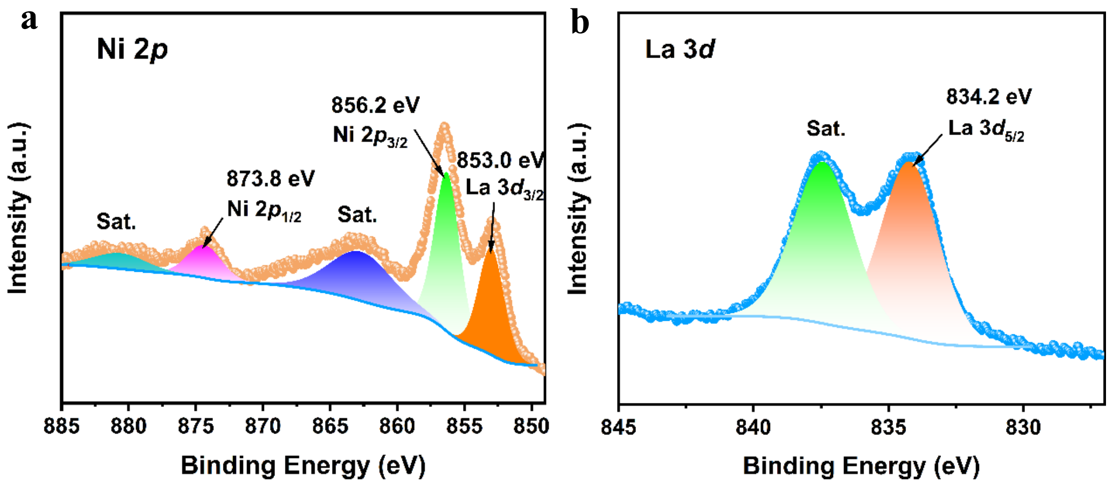

Supplementary Fig. 7 XPS spectra. XPS spectra of **a** Ni 2*p* and **b** La 3*d* in LaNi-Phen/COF-5. The high-resolution XPS spectrum of Ni 2*p* peaks located at 856.2 and 873.8 eV may be indexed to Ni $2p_{3/2}$ and Ni $2p_{1/2}$, respectively, indicating the successful loading of Ni^{2+} ions into COF-5 colloid through the coordination of N atoms rather than metal cluster state[6]. And two peaks at 834.2 and 837.5 eV, which may be assigned to the signals from La $3d_{5/2}$ and the corresponding satellite peaks of La^{3+}, respectively[7].

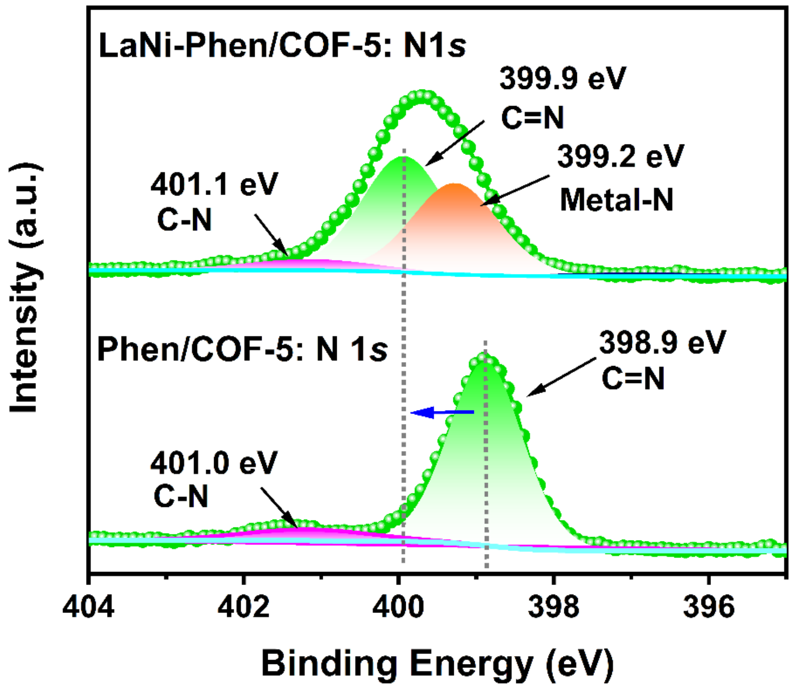

Supplementary Fig. 8 XPS spectra. XPS spectra of N 1*s* in COF-5 colloid and LaNi-Phen/COF-5, where the peak shift is significant, indicating strong interactions between La/Ni and the N atoms of 1,10-Phenanthroline (Phen) ligand.

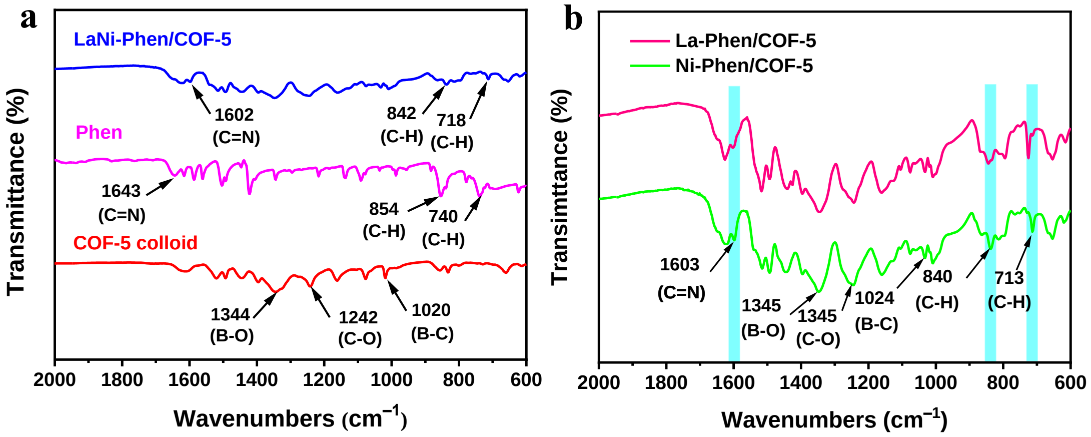

Supplementary Fig. 9 FTIR spectra. a FTIR spectra of COF-5 colloid, Phen and LaNi-Phen/COF-5. **b** FTIR spectra of La-Phen/COF-5 and Ni-Phen/COF-5, supporting that Ni-N and La-N bonds between $LaCl_3·6H_2O/NiCl_2·6H_2O$ and Phen groups are formed.

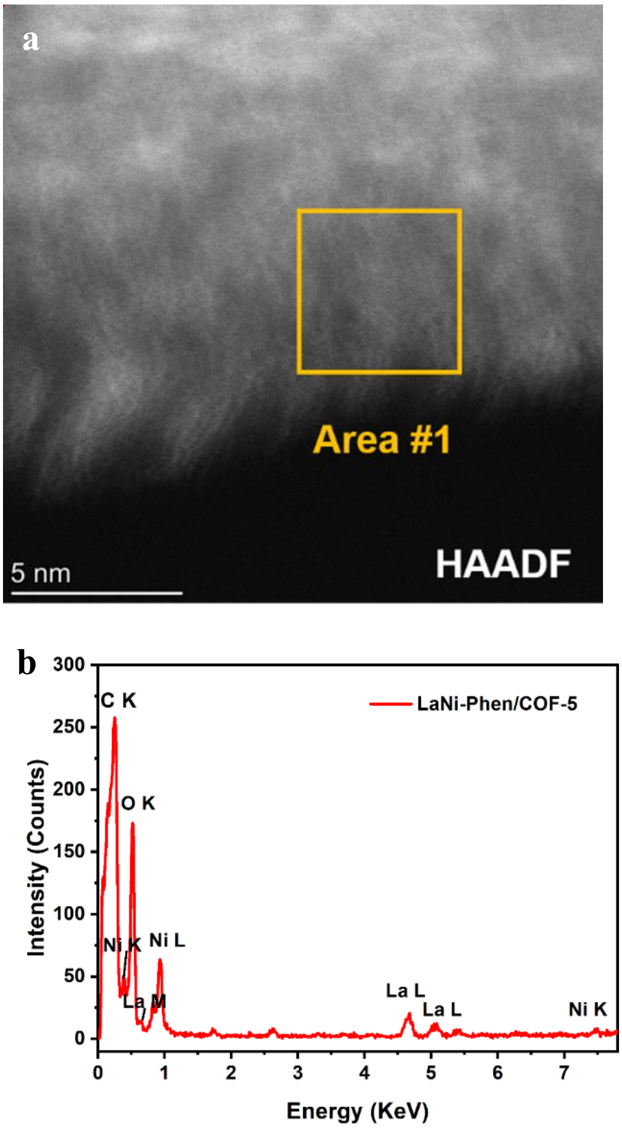

Supplementary Fig. 10 EDS spectra. a HAADF-STEM image of LaNi-Phen/COF-5.

b Corresponding EDX spectrum from the region indicated in HAADF-STEM image.

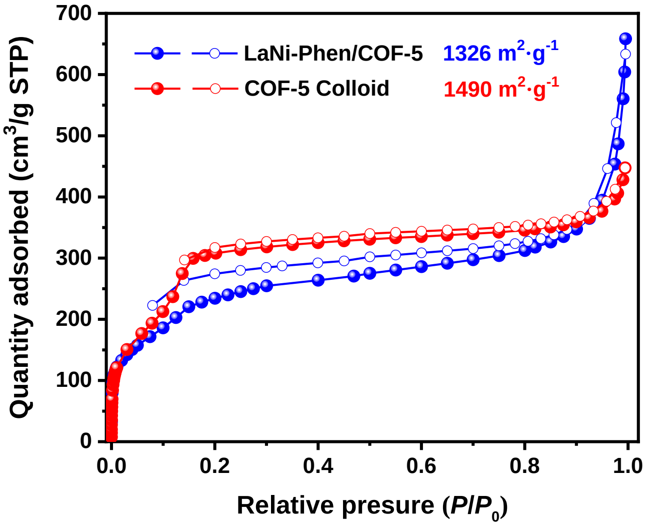

Supplementary Fig. 11 N_2 sorption isotherms. N_2 sorption isotherms of COF-5 colloid and LaNi-Phen/COF-5 at 77 K, suggesting that the incorporating of La and Ni single-sites into COF-5 colloid leads to a decreased BET surface area from 1490 to 1326 $m^2·g^{-1}$.

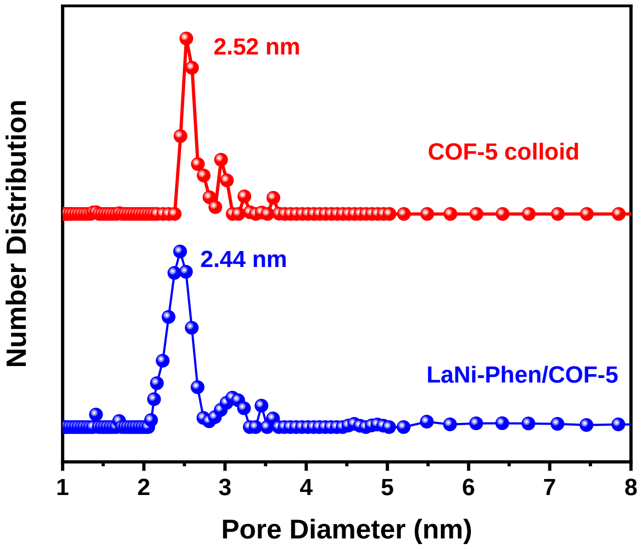

Supplementary Fig. 12 Pore size distribution. The calculated pore size distributions of COF-5 colloid and LaNi-Phen/COF-5. The pore size distributions of COF-5 colloid and LaNi-Phen/COF-5 are 25.2 Å and 24.4 Å, respectively, which are close to the

theoretical pore size of 27.2 Å for the simulated AA stacking model structure.

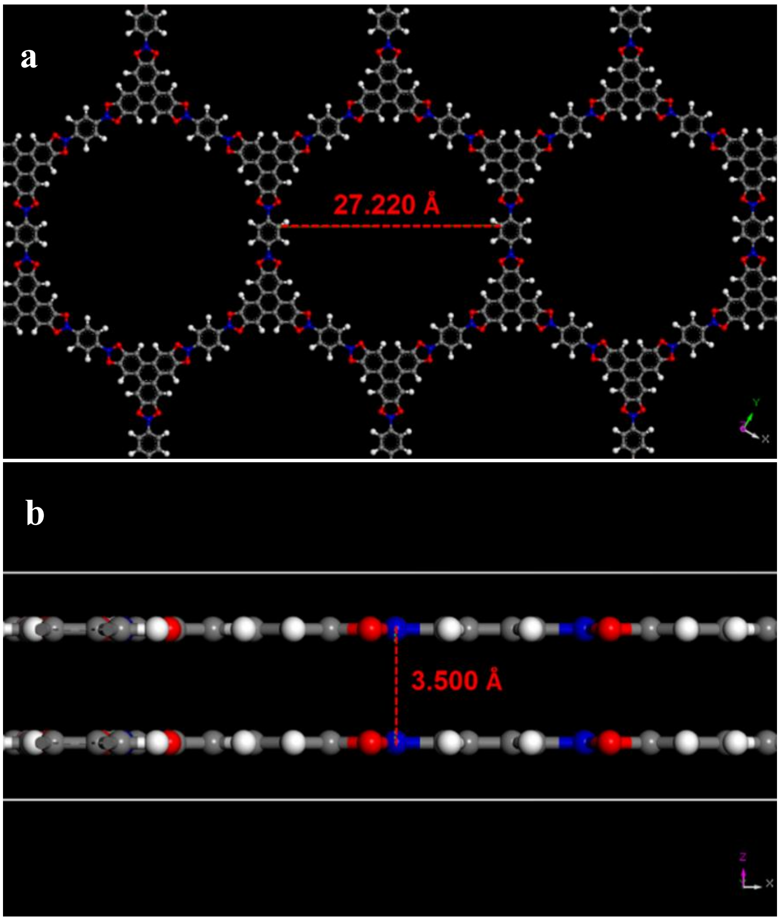

Supplementary Fig. 13 Molecular model of COF-5. a COF-5 view parallel to the z-axis. **b** COF-5 view parallel to the y-axis. The structural model calculated shows that the pore size and interlayer spacing of COF-5 are approximately 27.220 Å and 3.5 Å, respectively. The appropriate pore structure conduces to confine metal complexes and improve the CO_2 adsorption capacity. Carbon, oxygen, boron and hydrogen atoms are colored in grey, red, blue and white, respectively.

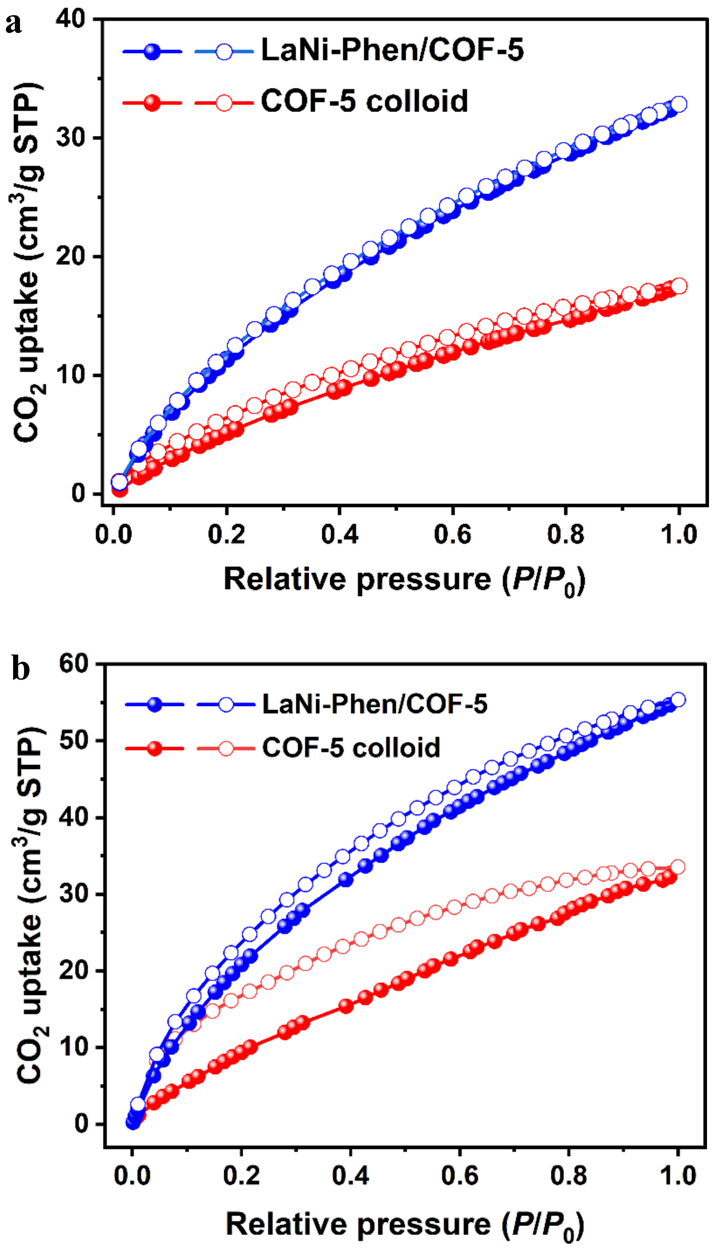

Supplementary Fig. 14 CO_2 adsorption curves. CO_2 adsorption isotherms of COF-5 colloid and LaNi-Phen/COF-5 at **a** 273 K and **b** 298 K, indicating the excellent CO_2 capture capability of LaNi-Phen/COF-5 as compared to COF-5 colloid.

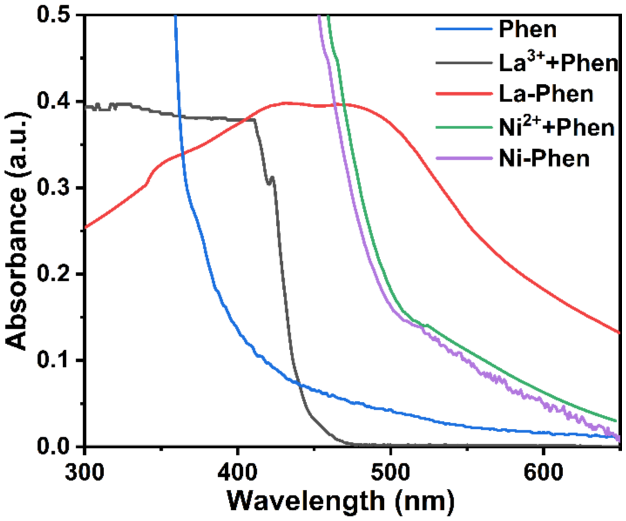

Supplementary Fig. 15 Absorption properties. UV-vis diffuse reflectance spectra of Phen, Ni^{2+}+Phen (physical mixing), Ni-Phen, La^{3+}+Phen (physical mixing) and La-Phen, proving that La-Phen acts as the light harvesting center determining the absorption range of the functional COF-5 colloids.

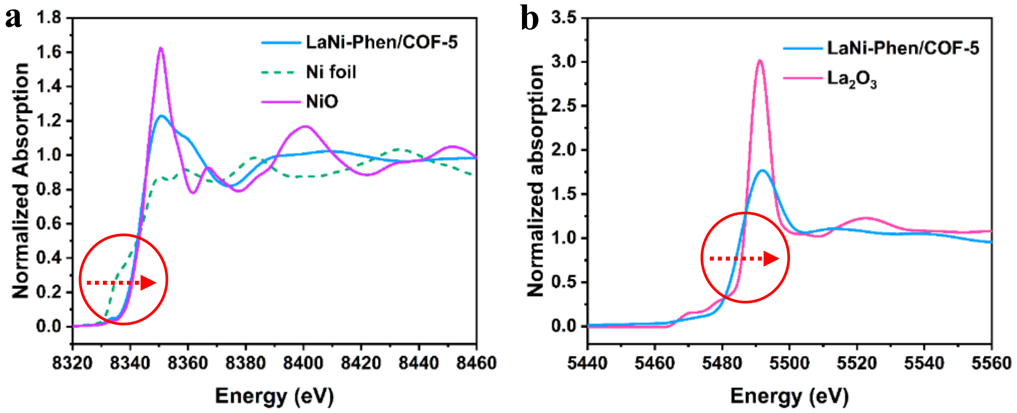

Supplementary Fig. 16 Normalized XANES spectra. a Experimental K-edge XANES spectra of Ni. **b** Experimental L3-edge XANES spectra of La. As highlighted with red circles and arrows, the near-edge absorption energy of La/Ni species in LaNi-Phen/COF-5 locates between that of the metal and the metal oxide, proving the positive charge of the La and Ni species.

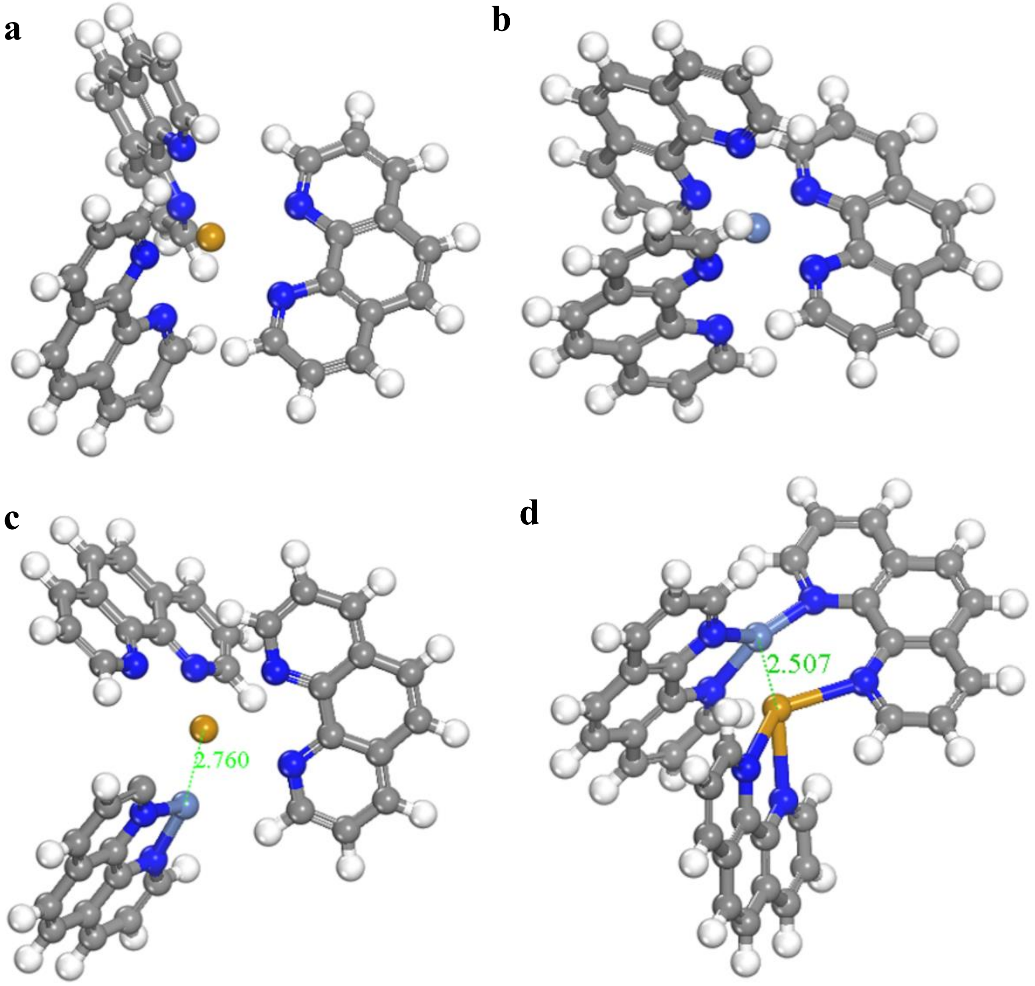

Supplementary Figure 17: Simulated local structure of **a** La-$(Phen)_3$, **b** Ni-$(Phen)_3$, **c** La-N_4/Ni-N_2, **d** La-N_3/Ni-N_3.

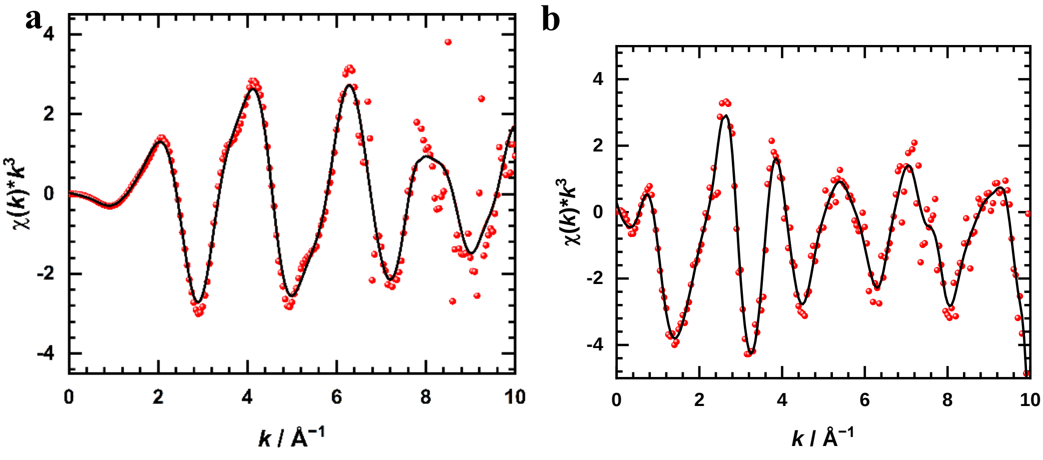

Supplementary Fig. 18 EXAFS fitting curves. Experimental EXAFS data (points) and curve fit (line) at *k* space of **a** Ni sites and **b** La sites.

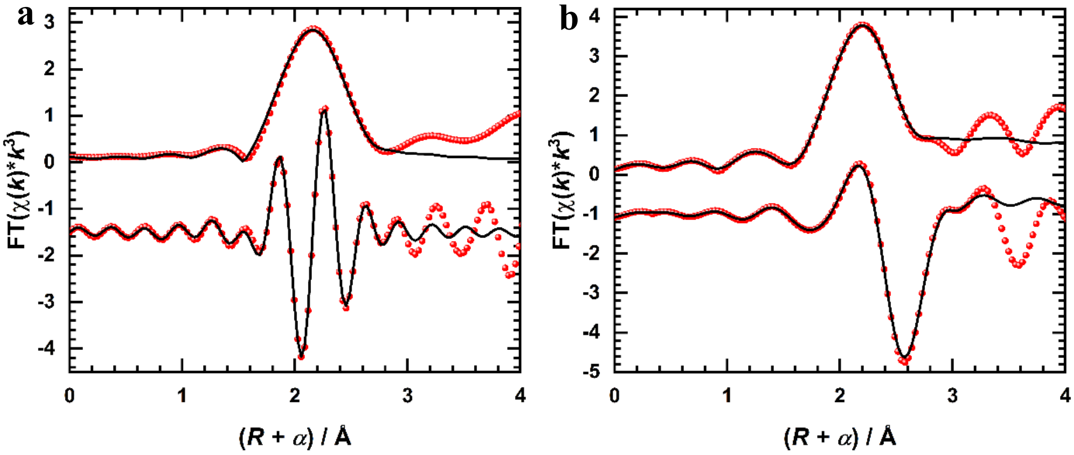

Supplementary Fig. 19 EXAFS fitting curves. Experimenetal EXAFS data (points) and curve fit (line) at *R* space (FT magnitude and imaginary component) of **a** Ni foil and **b** La_2O_3. The data are k^3-weighted and not phase-corrected.

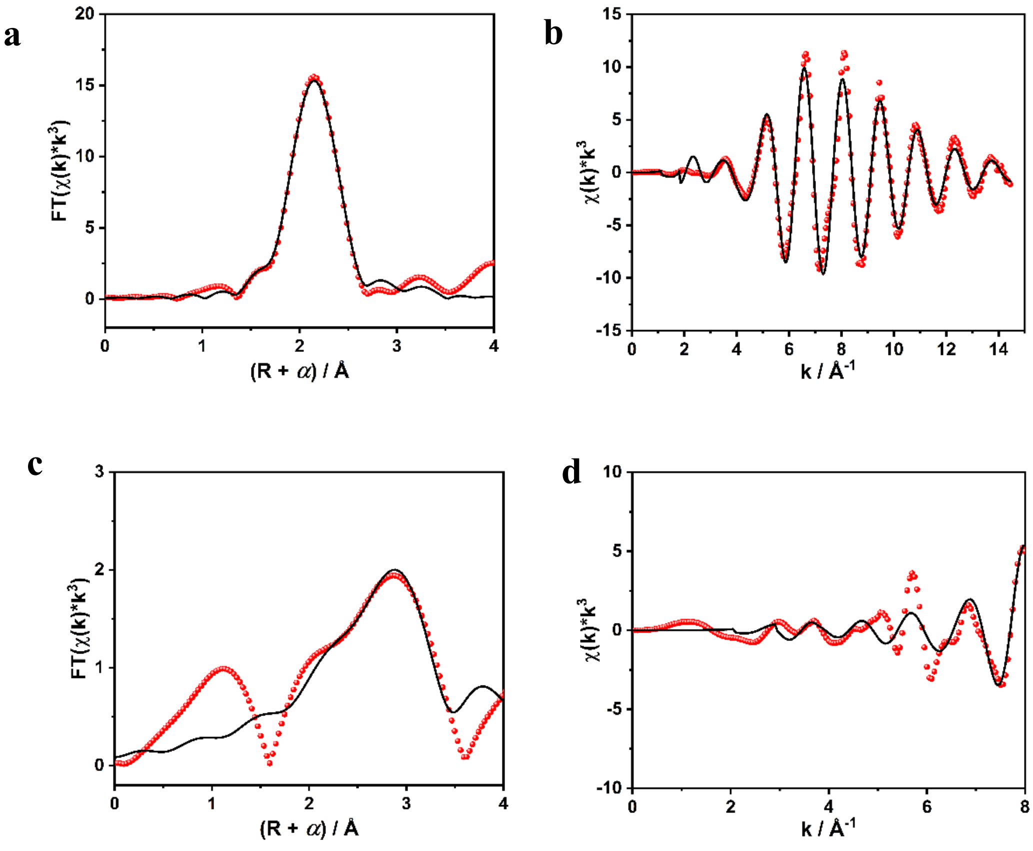

Supplementary Fig. 20 EXAFS fitting curves. Experimental EXAFS data (points) and curve fit (line) at *R* space (FT magnitude and imaginary component) of **a** Ni sites and **c** La sites for $LaNi_5$. The data are k^3-weighted and not phase-corrected. Experimental EXAFS data (points) and curve fit (line) at *k* space of **b** Ni sites and **d** La sites for $LaNi_5$.

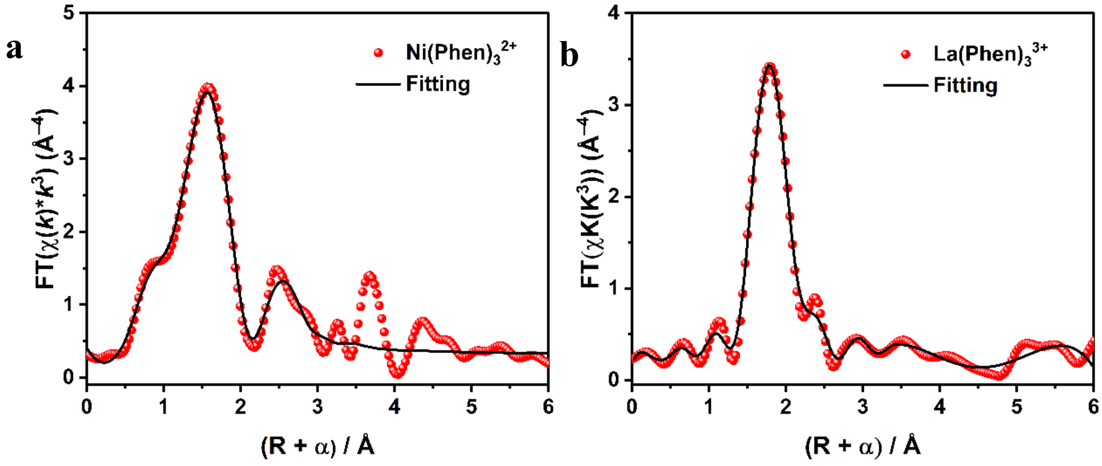

Supplementary Fig. 21 EXAFS fitting curves. Experimental EXAFS data (points) and curve fit (line) at *R* space (FT magnitude and imaginary component) of **a** $Ni(Phen)_3^{2+}$ and **b** $La(Phen)_3^{3+}$. The data are k^3-weighted and not phase-corrected.

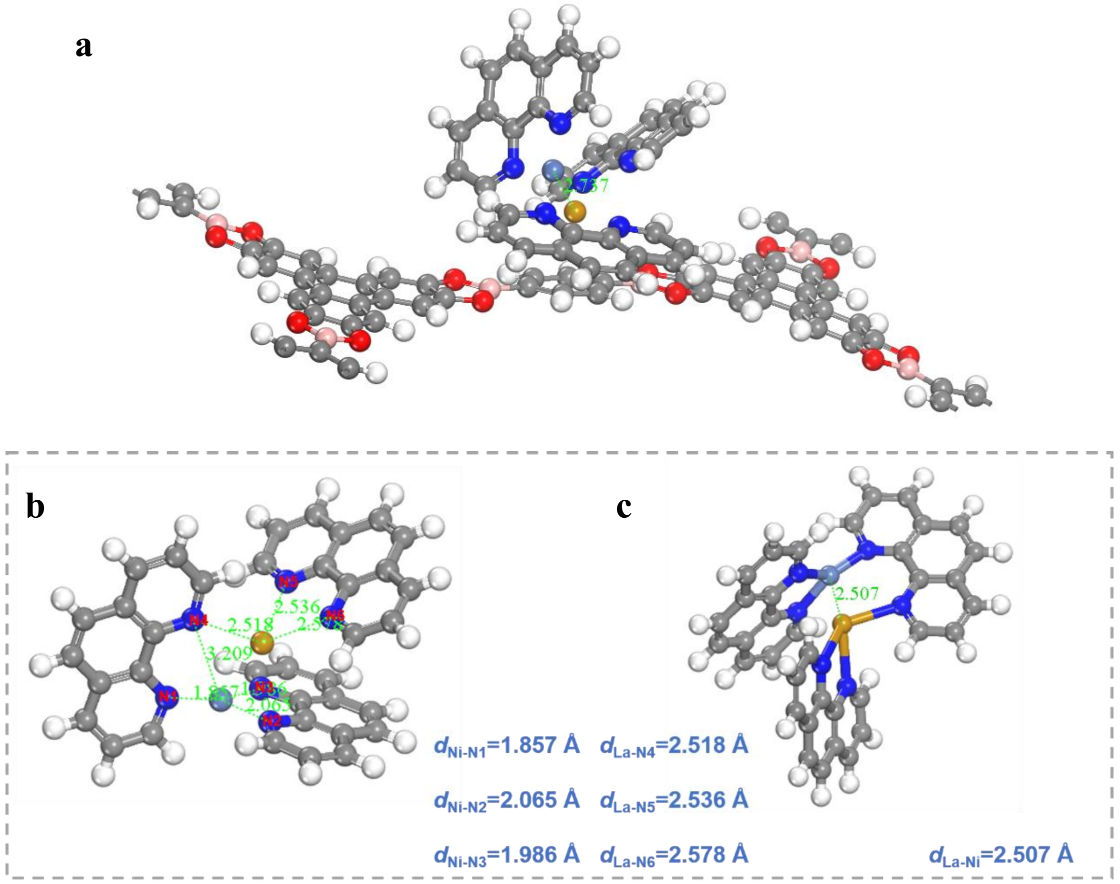

Supplementary Fig. 22 Simulated local structure of LaNi-Phen. a DFT-optimized structure of LaNi-Phen/COF-5, featuring that La and Ni are chelated by Phen with the coordinated structure of Ni-3N and La-3N. Schematic diagrams of **b** $d_{Ni\text{-}N}$, $d_{La\text{-}N}$ and **c** $d_{La\text{-}Ni}$. Color code: carbon-grey, oxygen-red, boron-pink, hydrogen-white, nitrogen-blue, nickel-light blue, lanthanum-yellow.

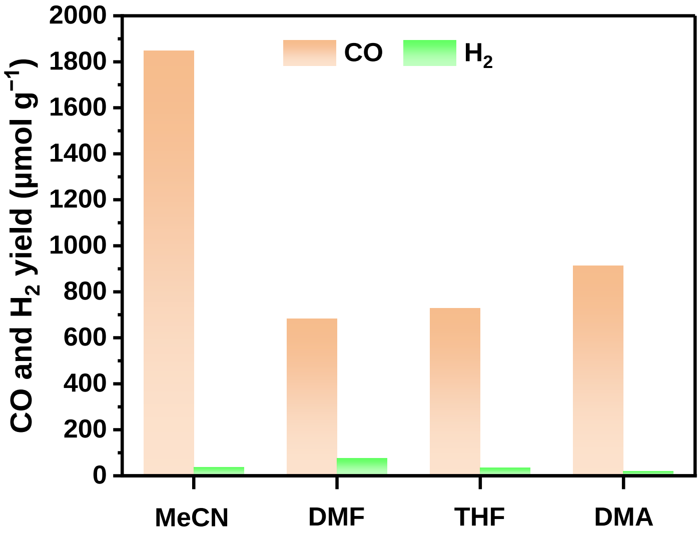

Supplementary Fig. 23 Effect of solvents in the CO_2 photoreduction. Effect of solvents in the CO_2 reduction over LaNi-Phen/COF-5 in a 3 h reaction, proving that acetonitrile is the most favorable reaction solvent with a higher activity and selectivity than the other solvents tested. The solution choice in a liquid-phase photocatalytic reaction plays a crucial role in CO_2 solubility and catalyst dispersion, which directly influences the catalytic efficiency. The results suggest that MeCN is the most suitable one. (Conditions: 10 mg photocatalysts, 10 mM BIH, 50 mL solvents, 2 mL H_2O, 300 W Xe lamp, 298 K).

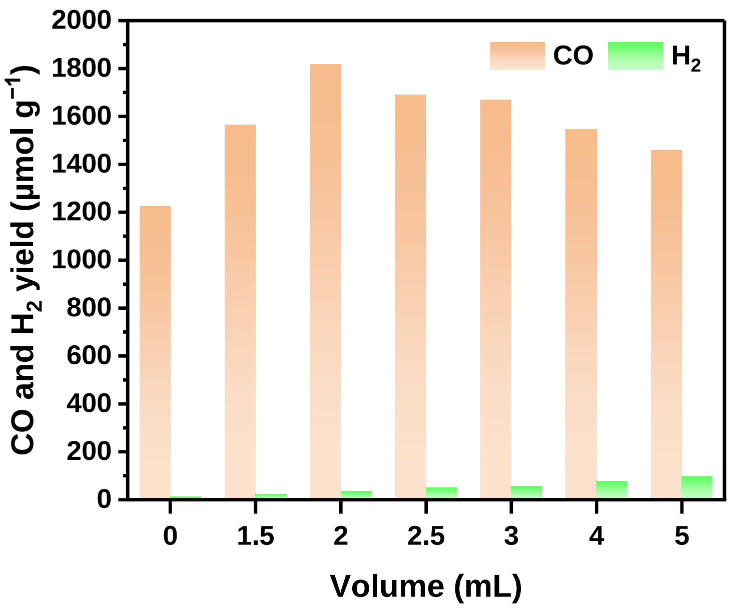

Supplementary Fig. 24 Effect of the H_2O content in the CO_2 reduction. Effect of the H_2O content in the CO_2 reduction over LaNi-Phen/COF-5 in a 3 h reaction, proving that H_2O significantly influences the catalytic activity and product selectivity. H_2O is the most commonly used proton donor. However, a competing reaction, which H_2O is reduced to H_2, may influence the CO_2 reduction process and then the product selectivity. In order to obtain a highly selective and active photocatalytic reaction system, the influence of the proton source, that is the volume of H_2O in the present work, should not be ignored. The results reveal that 2 mL H_2O in the reaction possesses the best performance. (Conditions: 10 mg photocatalysts, 10 mM BIH, 50 mL MeCN, *x* mL H_2O, 300 W Xe lamp, 298 K).

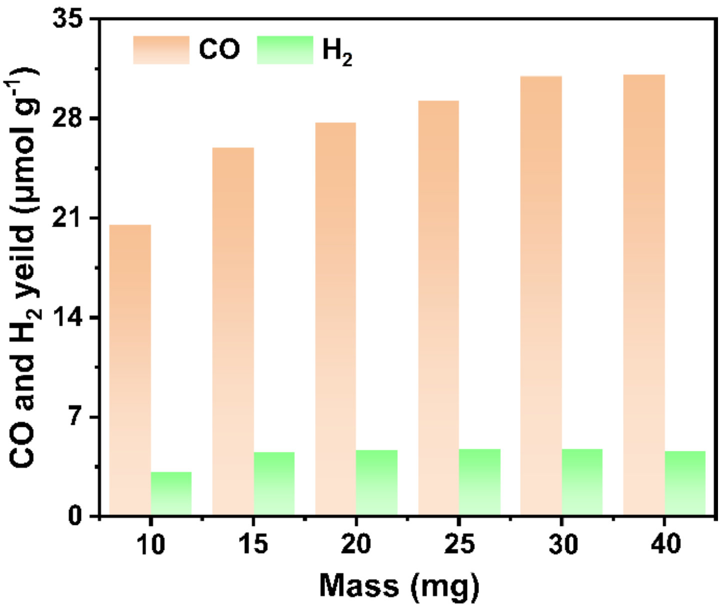

Supplementary Fig. 25 Effect of the weight of LaNi-Phen for CO_2 reduction. Effect of the weight of LaNi-Phen for CO_2 reduction in 3 h reaction, proving that confinement effect of COF-5 colloid on LaNi-Phen significantly influences the catalytic activity and product selectivity. The horizontal coordinate is the mass of the added photocatalyst. (Conditions: 10 mM BIH, 50 mL MeCN, *x* mL H_2O, 300 W Xe lamp, 298 K).

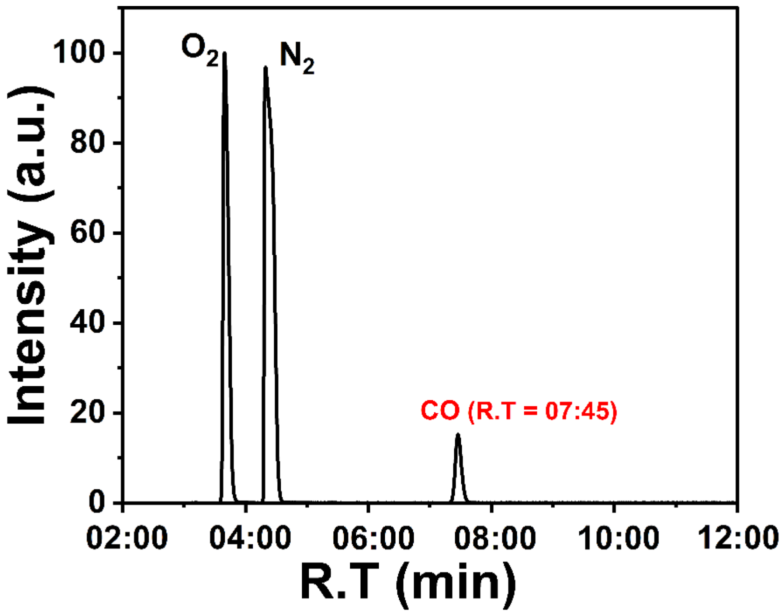

Supplementary Fig. 26 Gas chromatography-mass spectrometry for ^{13}C isotope tracer tests. Total ion chromatography in the photocatalytic reduction of $^{13}CO_2$ over LaNi-Phen/COF-5.

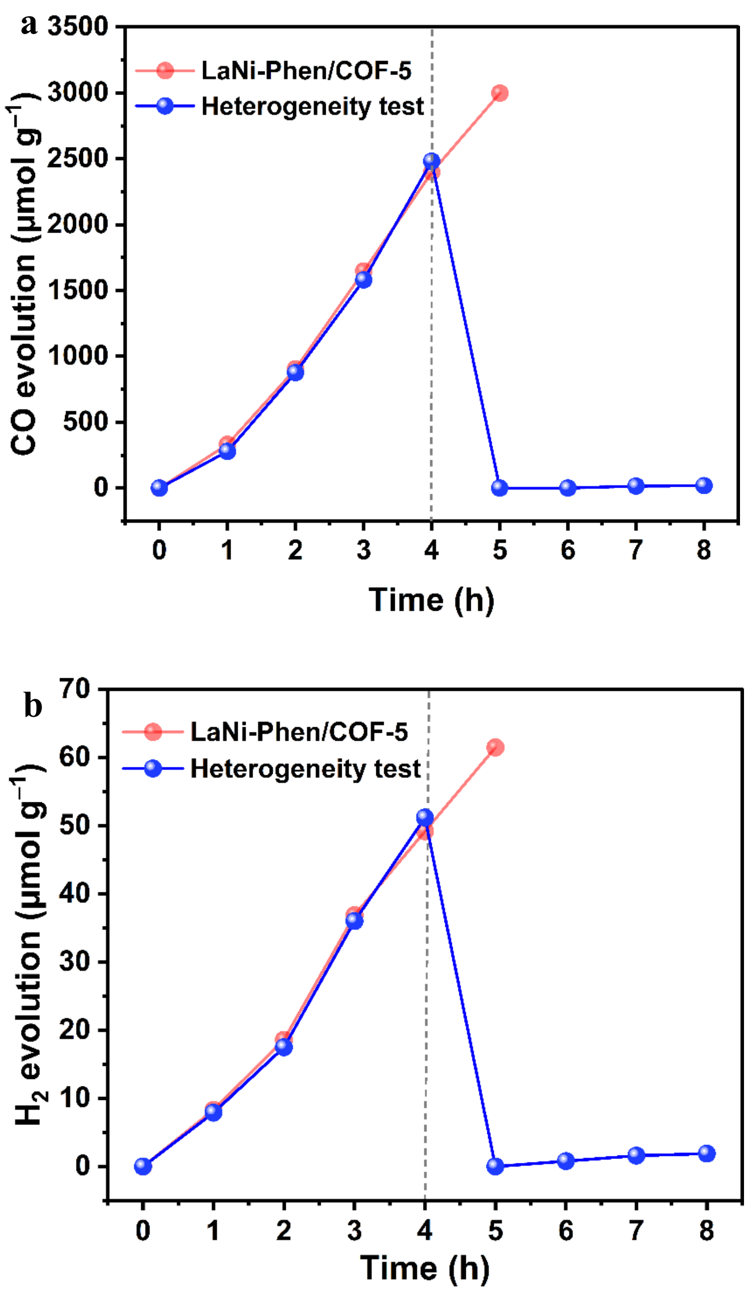

Supplementary Fig. 27 Heterogeneity tests. Effect of catalyst removal on the photocatalytic CO_2 reduction performance of **a** CO and **b** H_2 over LaNi-Phen/COF-5.

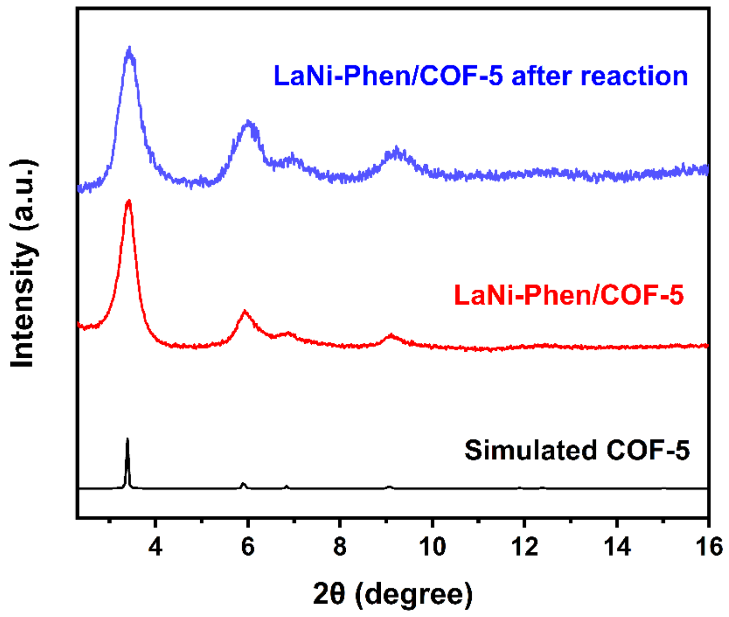

Supplementary Fig. 28 Powder XRD patterns. Powder XRD patterns of LaNi-Phen/COF-5 before and after CO_2RR reaction, evidencing the unaltered crystallinity after the CO_2RR process.

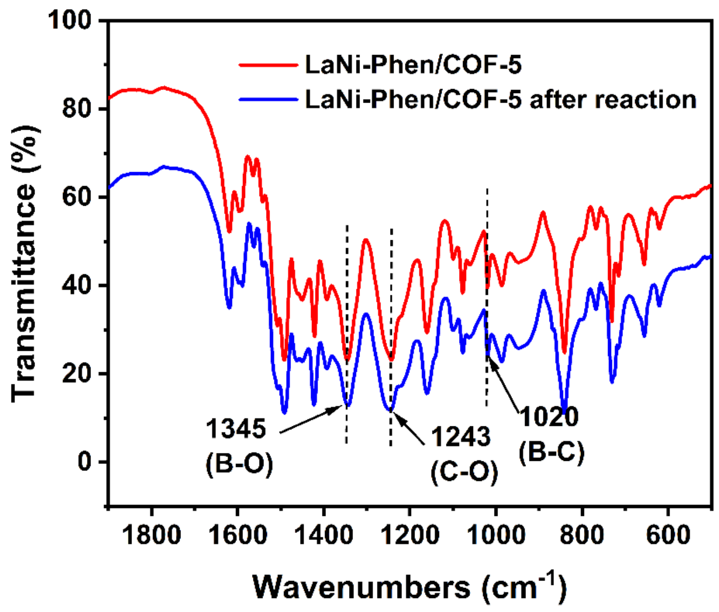

Supplementary Fig. 29 FTIR spectra. FTIR spectra of LaNi-Phen/COF-5 before and after CO_2RR reaction, showing the maintained integrity of the COF-5 colloidal framework structure after the CO_2RR process.

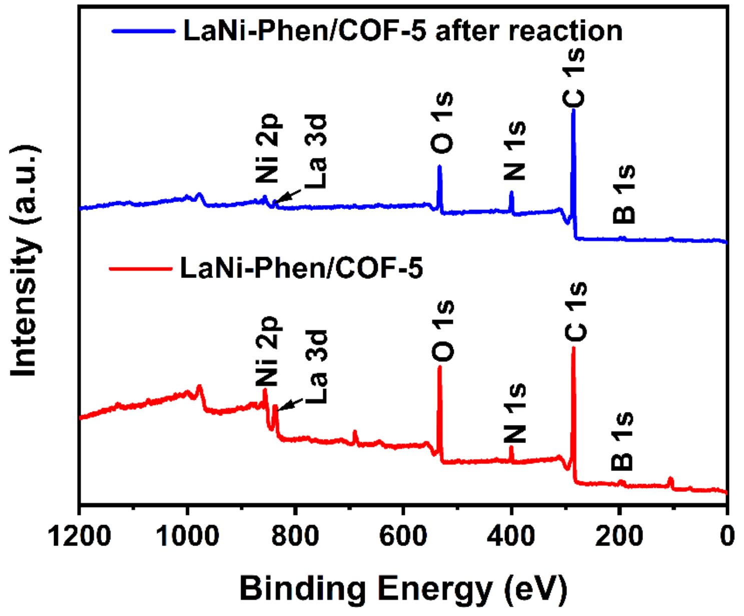

Supplementary Fig. 30 XPS spectra after CO_2RR reaction. XPS spectra of LaNi-Phen/COF-5 before and after the CO_2RR reaction (five 3 h-cycles).

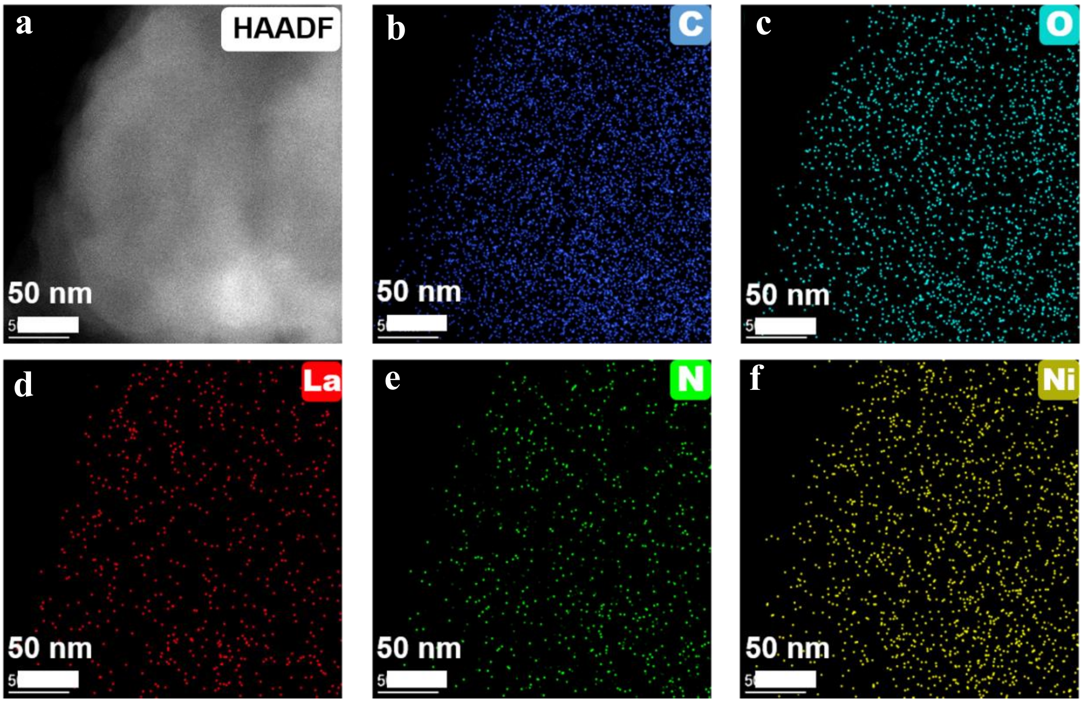

Supplementary Fig. 31 Morphology characterization after CO_2RR cycle experiments. a HAADF image, **b-f** separated elemental mapping images of LaNi-Phen/COF-5 after the CO_2RR reaction, evidencing the colocalization of the different elements without segregation or aggregation.

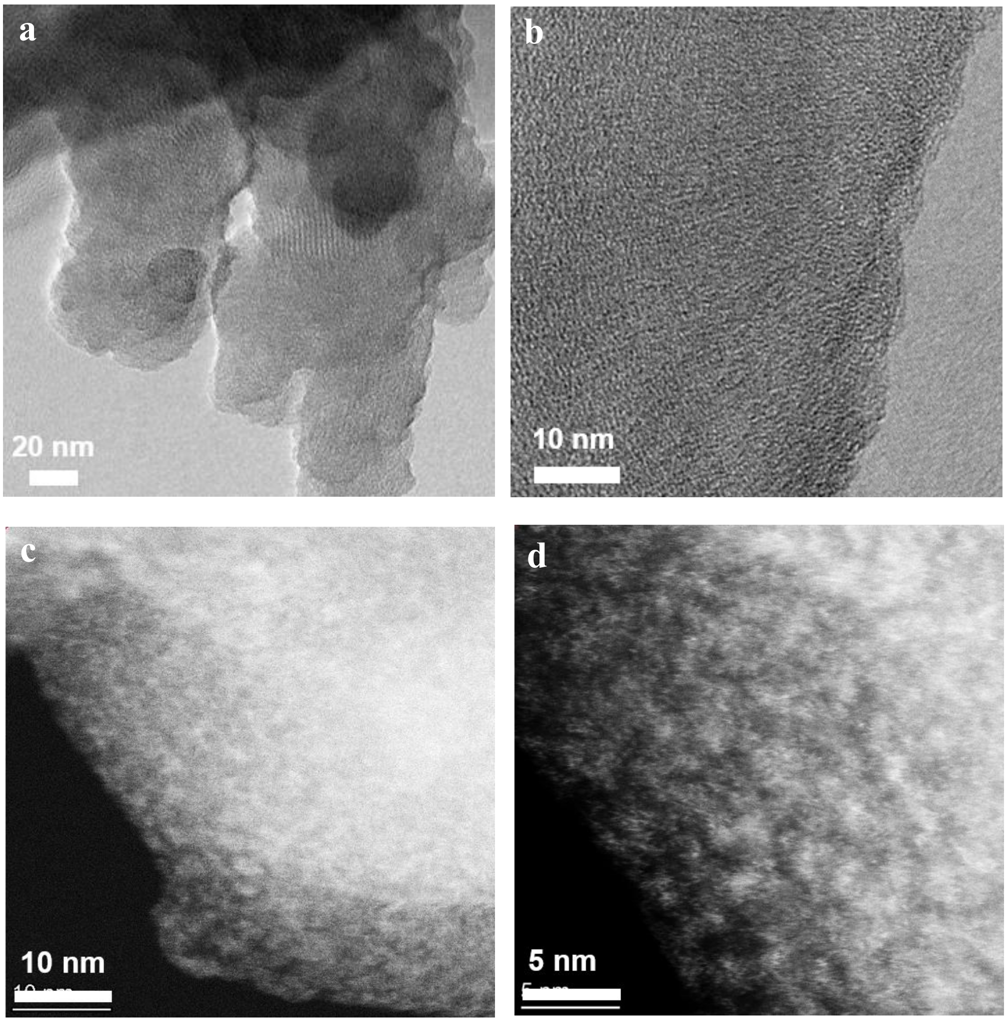

Supplementary Fig. 32 Morphology characterization after CO_2RR cycle experiments. a / b TEM images, **c / d** atomic-resolution ADF-STEM images of LaNi-Phen/COF-5 after CO_2RR reaction.

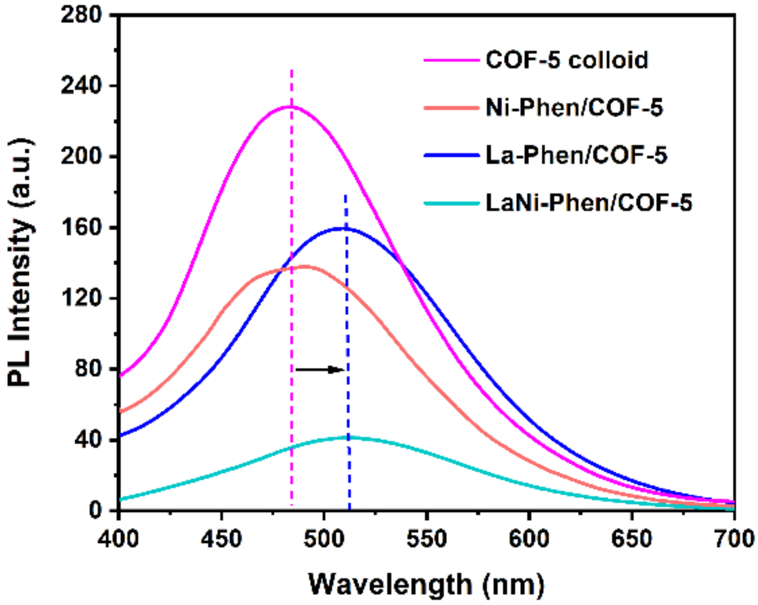

Supplementary Fig. 33 Steady-state PL spectra. Steady-state PL spectra of COF-5 colloid, Ni-Phen/COF-5, La-Phen/COF-5 and LaNi-Phen/COF-5 using an excitation wavelength of λ = 365 nm[8]. Compared with the COF-5 colloid, the PL peak position of LaNi-Phen/COF-5 is slightly red-shifted by around 26 nm (Supplementary Fig. 30), which is in accordance with the metal ion-induced reduction of the bandgap of COF-5 observed in the aforementioned UV-vis absorption studies (Supplementary Note 6). The lowest PL intensity of LaNi-Phen/COF-5 in this comparison also suggests its highest charge transport property.

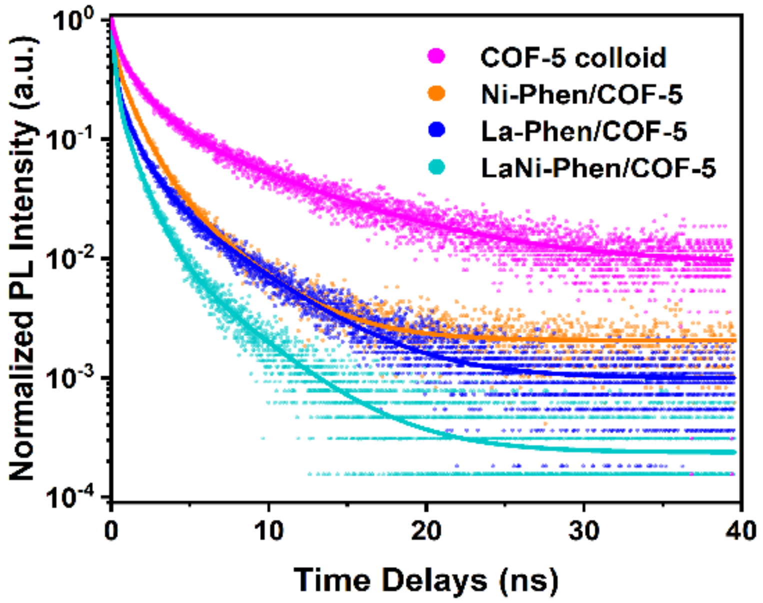

Supplementary Fig. 34 Transient-state PL spectra. Transient-state PL spectra of COF-5 colloid, Ni-Phen/COF-5, La-Phen/COF-5 and LaNi-Phen/COF-5 using an excitation wavelength of λ=365 nm.

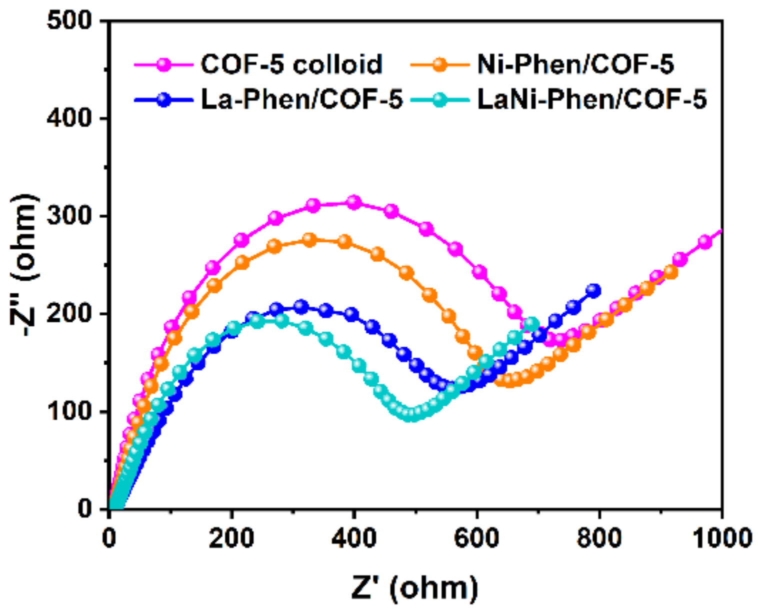

Supplementary Fig. 35 Electrochemical impedance spectra. Electrochemical impedance spectra of COF-5 colloid, Ni-Phen/COF-5, La-Phen/COF-5 and LaNi-Phen/COF-5.

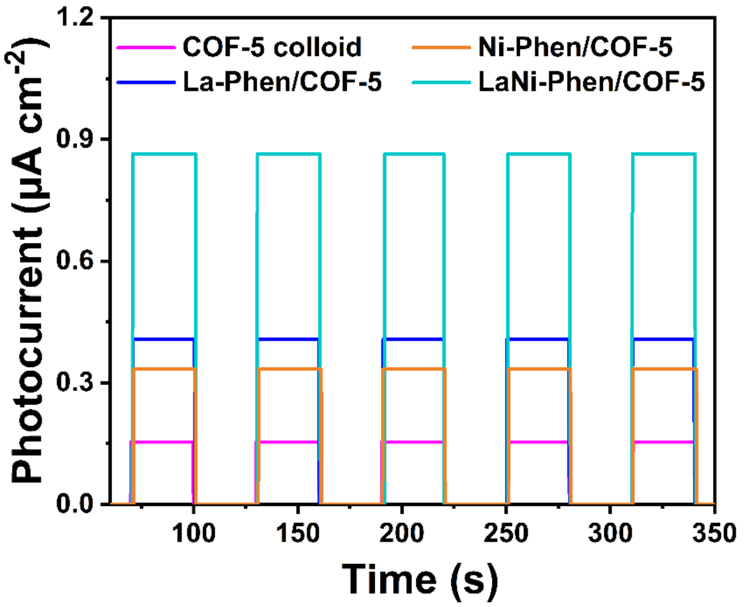

Supplementary Fig. 36 Photocurrent-time (I-t) responses. Photocurrent-time (I-t) responses of COF-5 colloid, Ni-Phen/COF-5, La-Phen/COF-5 and LaNi-Phen/COF-5.

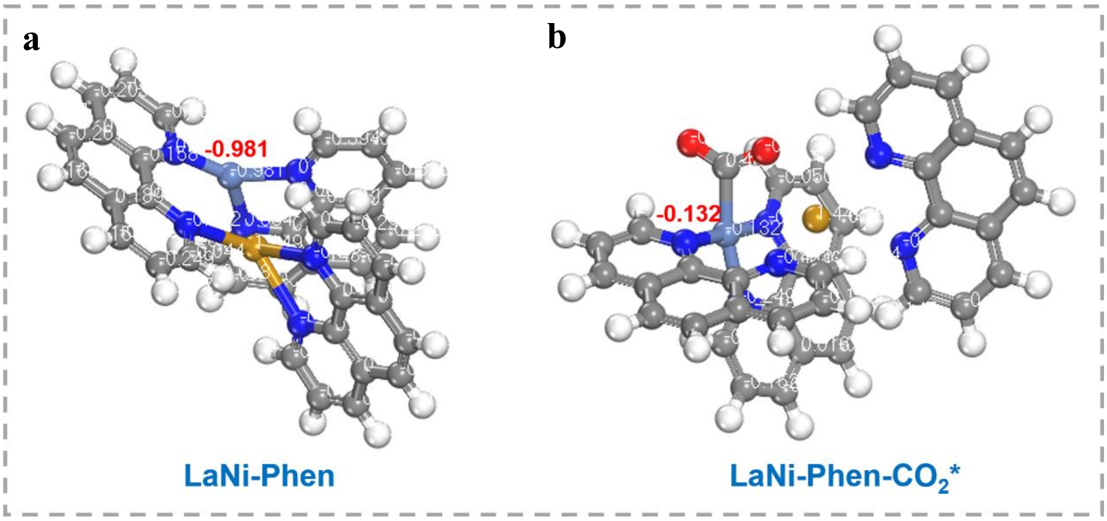

Supplementary Fig. 37 Surface charge calculation. a Surface charge of Ni atoms and **b** surface charge of Ni atoms after adsorption of CO_2.

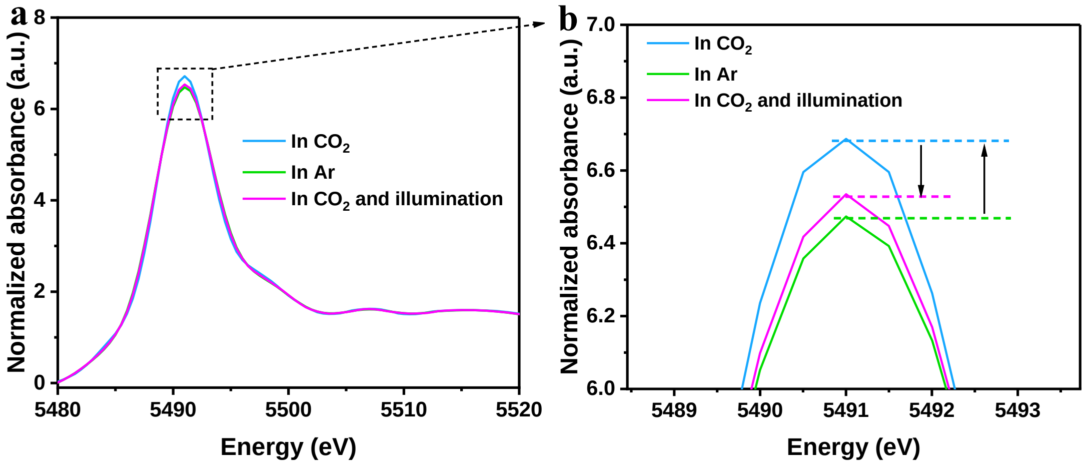

Supplementary Fig. 38 In situ XAFS measurements. a La L3-edge XANES spectra of LaNi-Phen/COF-5 during the CO_2 photoreduction reaction at room temperature, in 1 atm of Ar or CO_2-saturated aqueous solution. **b** Enlarged La L3-edge XANES spectra, demonstrating that La atoms also act as catalytic centers, and that the electronic structure is closely correlated with the processes of adsorption, activation, and the photocatalytic CO_2RR.

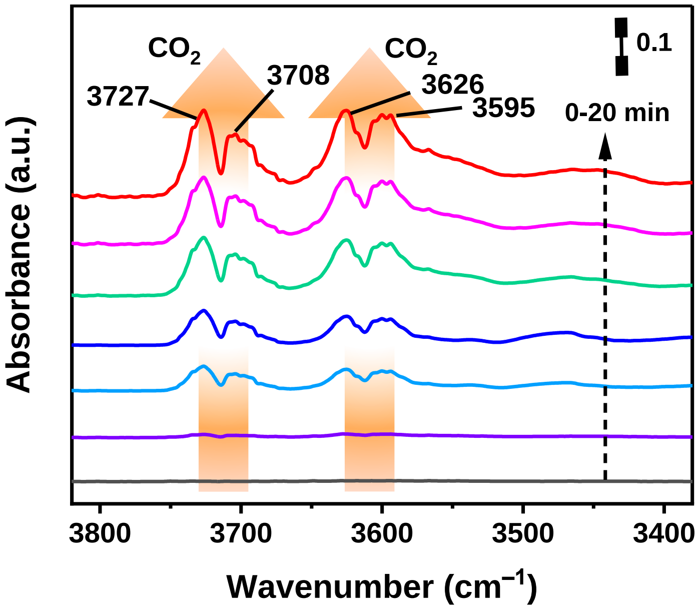

Supplementary Fig. 39 In situ DRIFTS spectra test. In-situ DRIFTS spectra of the CO_2 interaction with LaNi-Phen/COF-5 in the dark.

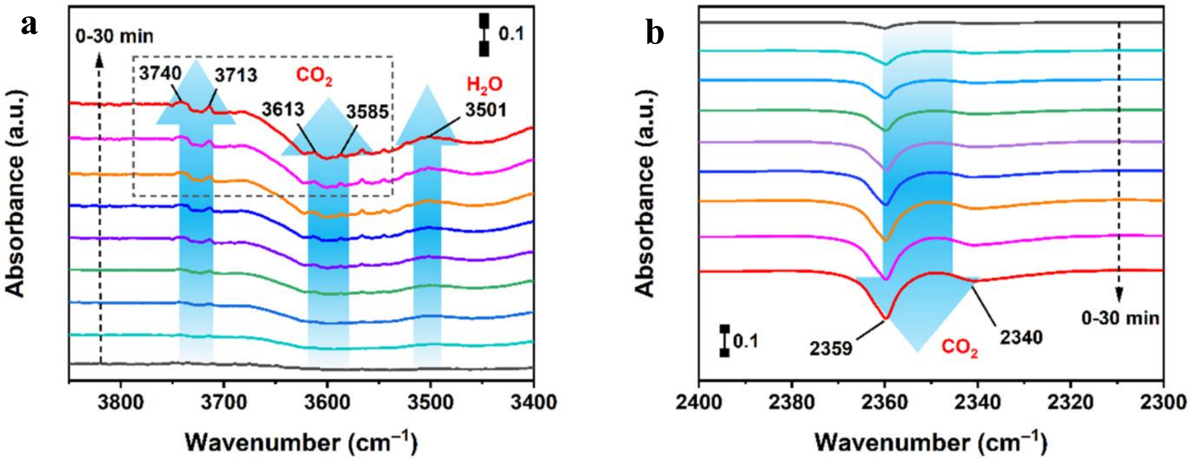

Supplementary Fig. 40 In-situ DRIFTS spectra test, CO_2 and H_2O interaction with LaNi-Phen/COF-5 under subsequent light irradiation. a Intensities of adsorbed gas-phase CO_2 at 2800-3800 cm^{-1}. **b** Intensities of $v_3(CO_2)$ absorption of surface CO_2 molecules at 2300-2400 cm^{-1}.

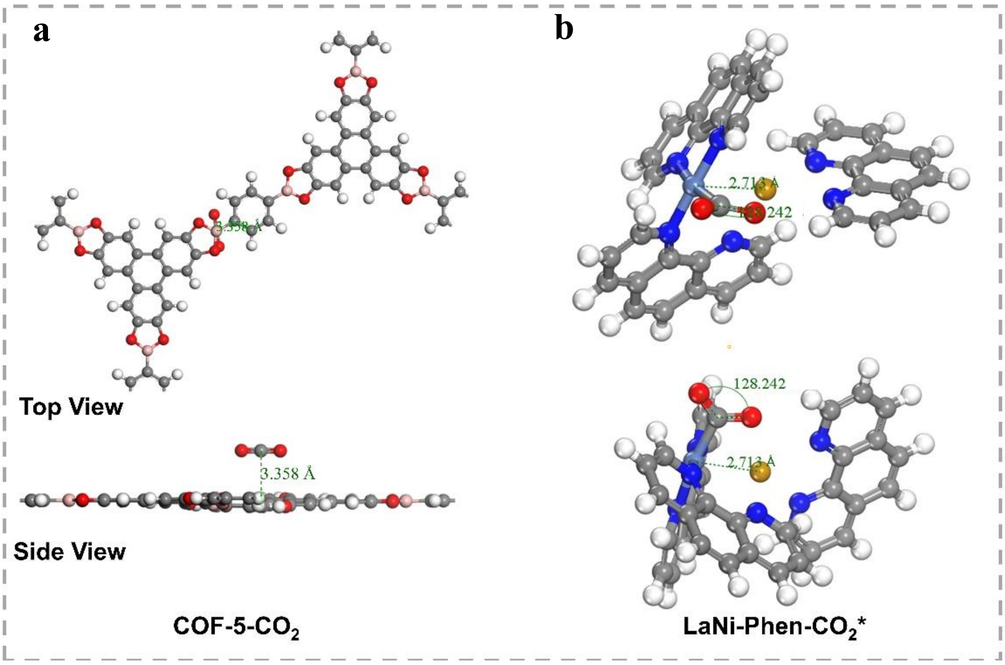

Supplementary Fig. 41 DFT simulation of CO_2 adsorption. DFT simulation of the CO_2 adsorption over the COF-5 surface **a**, and LaNi-Phen **b**. The O=C=O bond on the COF-5 surface was in a linear configuration, indicating that sole COF-5 cannot effectively activate the CO_2 molecule. Notably, the O=C=O bond on the LaNi-Phen coordination configuration evolves into a V-type configuration with a bond angle of 128.24°, which fully verified that the La-N_3/Ni-N_3 configuration is beneficial to the adsorption and activation of CO_2.

Supplementary Tables

Supplementary Table 1 Elemental composition of LaNi-Phen/COF-5. Elemental analysis using EDX showing the elemental contents of La and Ni in LaNi-Phen/COF-5 from (the copper signal comes from the copper grid).

Element (edge)	Weight%	Atomic%	Net Error%
C (K)	60.56	74.39	4.85
N (K)	2.56	2.17	0.59
O (K)	18.54	16.92	3.43
Cl (K)	1.95	0.91	0.19
Cu (K)	12.20	3.18	0.18
La (L)	2.58	1.53	0.21
Ni (K)	1.61	0.90	0.15

Supplementary Table 2 EXAFS fitting parameters. EXAFS curve fitting parameters[a] for the Ni K-edge and the La L3-edge for different samples.

Sample	Path	*N*	R / Å	$\sigma^2/Å^2$	ΔE_0 (eV)	R-factor/%
Ni foil	Ni–Ni[b]	12.0[f]	2.47	0.006	2.3	0.5
LaNi-Phen/COF-5	Ni–N[c]	3.0	1.98	0.004	2.6	0.6
LaNi-Phen/COF-5	Ni–N[c]	2.3	1.91	0.003	2.3	0.4
La_2O_3	La–O[d]	6.0[f]	2.57	0.005	4.5	0.4
LaNi-Phen/COF-5	La–N[e]	4.2	2.54	0.007	−3.1	0.2
LaNi-Phen/COF-5	La–N[e]	3.0	2.62	0.008	-3.3	0.3
$[La(Phen)_3]^{3+}$	La–N[e]	6.0	2.56	0.004	2.6	0.3
$[Ni(Phen)_3]^{2+}$	Ni–N[e]	6.0	2.13	0.005	−2.8	0.5

[a]*N*, coordination number; R, distance between absorber and backscatter atoms; σ^2, Debye-Waller factor to account for both thermal and structural disorders; ΔE_0, inner potential correction; R factor (%) indicates the goodness of the fit. The accuracies of the above parameters were estimated as N, ±20%; R, ±1%; σ^2 ±20%; ΔE_0, ±20%. For Ni foil, La_2O_3, and LaNi-Phen/COF-5 samples, S_0^2 was determined from Ni foil (0.875), La_2O_3 (0.907), and Porphyrin-Cu (II) (0.853) reference fitting. [b]Fitting range: $2.5 \leq k \leq 14.5$ Å^{-1}, $1.0 \leq R \leq 3.0$ Å. [c]Fitting range: $0 \leq k \leq 10$ Å^{-1}, $1.0 \leq$ R (Å) ≤ 3.0 Å. [d]Fitting range: $2.5 \leq k \leq 10.0$ Å^{-1}, $1.0 \leq R \leq 3.0$ Å. [e]Fitting range: $0 \leq k \leq 10.0$ Å^{-1}, $1.0 \leq R \leq 3.0$ Å. [f]These coordination numbers were constrained as *N*(Ni-Ni) = 12.0 and *N*(La-O) = 6.0 based on the crystal structure.

Supplementary Table 3 Photocatalytic CO_2 reduction performance over La_xNi_{1-x}-Phen/COF-5 with various La-Ni metal ion doping proportions.

Entry[a]	Catalyst	Production rates (μmol·g^{-1}·h^{-1})		S_{CO} (%)[b]
		CO	H_2	
1	COF-5 colloid	39.9	16.5	70.7
2	Ni-Phen/COF-5	224.4	21.7	91.2
3	$La_{0.1}Ni_{0.9}$-Phen/COF-5	236.8	19.3	92.5
4	$La_{0.2}Ni_{0.8}$-Phen/COF-5	299.4	17.9	94.3
5	$La_{0.3}Ni_{0.7}$-Phen/COF-5	342.9	22.8	93.8
6	$La_{0.4}Ni_{0.6}$-Phen/COF-5	446.5	17.4	96.2
7	$La_{0.5}Ni_{0.5}$-Phen/COF-5	549.5	19.8	96.5
8	$La_{0.6}Ni_{0.4}$-Phen/COF-5	605.8	11.3	98.2
9	$La_{0.7}Ni_{0.3}$-Phen/COF-5	565.3	14.2	97.5
10	$La_{0.8}Ni_{0.2}$-Phen/COF-5	473.7	16.7	96.6
11	$La_{0.9}Ni_{0.1}$-Phen/COF-5	248.9	12.1	95.4
12	La-Phen/COF-5	195.4	8.2	95.9

[a]Reaction conditions: 10 mg catalyst and 30 mg (molar concentration of 2.4 mM) BIH as a sacrificial agent; 50 mL CH_3CN and 2 mL H_2O; UV-vis light irradiation (350 W Xenon lamp, $\lambda > 380$ nm), 1 atm CO_2 and 25 °C.

[b]Electron selectivity (***Sel****electron*) of CO: $S_{CO}(\%) = [2v(CO)]/[2v(CO) + 2v(H_2)] \times 100\%$, where v(CO) and $v(H_2)$ denote the formation rates of CO and H_2, respectively.

Supplementary Table 4 Performance comparison. LaNi-Phen/COF-5 was compared with other reported photocatalysts without the additional photosensitizers in terms of CO activity and selectivity.

Catalyst	Light source	CO activity ($\mu mol \cdot g^{-1} \cdot h^{-1}$)	CO selectivity	Ref
**LaNi-Phen/COF-5**	**300 W Xe lamp**	**605.8**	**98.2**	**This work**
Co_4/g-C_3N_4	300 W Xe lamp	89.6	93.4	9
TAPBB-COF	300 W Xe lamp	24.6	95.6	10
TTCOF-Zn	300 W Xe lamp	2.05	/	11
O/La-CN	300 W Xe lamp	92	80.4	12
Co-ZIF-9/TiO_2	300 W Xe lamp	17.58	90.0	13
NH2-UiO-66/$CsPbBr_3$	300 W Xe lamp	8.21	97.0	14
CT-COF	300 W Xe lamp	102.7	98.0	15
Ni-SA-x/ZrO_2	300 W Xe lamp	11.8	92.5	16
QA/10$BiVO_4$	300 W Xe lamp	407	87	17
CN/rGO	300 W Xe lamp	12.63	87.0	18
WO_3/CN	UV ($254 \leq \lambda < 420$nm)	58.40	/	19
Er/g-C_3N_4	Simulated solar irradiation	12.75	87.6	20
CoRu-HCNp	300 W Xe lamp	27.3	/	21
$LaPO_4$/g-C_3N_4	300 W Xe lamp	14.4	/	22
Ni/Mn-oxo/g-C_3N_4	300 W Xe lamp	13.9	62.3	23

Supplementary Table 5 Comparison of total electron transfer in different catalysts.

Catalyst[a]	Production rates ($\mu mol \cdot g^{-1} \cdot h^{-1}$)		Total electron transfer ($\mu mol \cdot g^{-1} \cdot h^{-1}$)
	CO	H_2	
**LaNi-Phen/COF-5**	**605.8**	**11.7**	**1235.0**
Ni-Phen/COF-5	224.4	21.7	492.2
La-Phen/COF-5	195.4	8.2	407.2
COF-5 colloid	39.9	16.5	112.8
mix-LaNi-Phen/COF-5	115.8	14.6	260.8
LaNi-Phen	22.4	12.8	70.4

*Total electron transfer=2×CO production rate+2×H_2 production rate.

Supplementary Table 6 Fitted TRPL parameters of COF-5 colloid, Ni-Phen/COF-5, La-Phen/COF-5 and LaNi-Phen/COF-5.

Sample	τ_1 (ns)	A_1 (%)	τ_2 (ns)	A_2 (%)	τ_3 (ns)	A_3 (%)	τ_{ave} (ns)
COF-5 colloid	0.39	44.38	1.67	41.11	7.49	14.51	4.75
Ni-Phen/COF-5	0.21	47.54	1.09	47.17	3.50	5.39	1.54
La-Phen/COF-5	0.17	78.71	1.02	19.50	4.21	1.83	1.34
LaNi-Phen/COF-5	0.12	82.92	0.19	15.06	1.88	2.04	0.53

*The lifetime fitted by the following triexponential equation: $y = A_1\exp(-t/\tau_1) + A_2\exp(-t/\tau_2) + A_3\exp(-t/\tau_3) + y_0$. The average lifetime is obtained by the following equation: $\tau_{ave} =(A_1\tau_1^2 +A_2\tau_2^2+A_3\tau_3^2)/(A_1\tau_1+A_2\tau_2+A_3\tau_3)$.

Supplementary References

1. Daniel, G. et al. Platinum-free electrocatalysts for oxygen reduction reaction: Fe-N_x modified mesoporous carbon prepared from biosources. *J. Power Sources* **402**, 434-446 (2018).
2. Serov, A., Artyushkova, K. & Atanassov, P. Fe-N-C oxygen reduction fuel cell catalyst derived from carbendazim: Synthesis, structure, and reactivity. *Adv. Energy Mater.* **4**, 1301735 (2014).
3. Cote, A. P., Benin, A. I., Ockwig, N. W., O'Keeffe, M., Matzger, A. J. & Yaghi, O. M. Porous, crystalline, covalent organic frameworks. *Science* **310**, 1166-1170 (2005).
4. Ma, X. P. et al. The intercalation of 1,10-phenanthroline into layered $NiPS_3$ via iron dopant seeding. *Chem. Commun.* **56**, 4603-4606 (2020).
5. Fang, Z. B. et al. Boosting interfacial charge-transfer kinetics for efficient overall CO_2 photoreduction via rational design of coordination spheres on metal-organic frameworks. *J. Am. Chem. Soc.* **142**, 12515-12523 (2020).
6. Zhong, W. F. et al. A covalent organic framework bearing single Ni sites as a synergistic photocatalyst for selective photoreduction of CO_2 to CO. *J. Am. Chem. Soc.* **141**, 7615-7621 (2019).
7. Liu, X. F. et al. Rare earth La single atoms supported MoO_{3-x} for efficient photocatalytic nitrogen fixation. *Appl. Catal. B-Environ.* **301**, 120766 (2022).
8. Evans, A. M. et al. Emissive single-crystalline boroxine-linked colloidal covalent organic frameworks. *J. Am. Chem. Soc.* **141**, 19728-19735 (2019).
9. Zhou, J. et al. Oxidative polyoxometalates modified graphitic carbon nitride for visible-light CO_2 reduction. *ACS Appl. Mater. Interfaces* **9**, 11689-11695 (2017).
10. Wang, L. J., Wang, R. L., Zhang, X., Mu, J. L., Zhou, Z. Y. & Su, Z. M. Improved photoreduction of CO_2 with water by tuning the valence band of covalent organic frameworks. *ChemSusChem* **13**, 2973-2980 (2020).
11. Lu, M. et al. Rational design of crystalline covalent organic frameworks for efficient CO_2 photoreduction with H_2O. *Angew. Chem. Int. Ed.* **58**, 12392-12397

(2019).

12. Chen, P. et al. Rare-earth single-atom La-N charge-transfer bridge on carbon nitride for highly efficient and selective photocatalytic CO_2 reduction. *ACS Nano* **14**, 15841-15852 (2020).
13. Yan, S. S., Ouyang, S. X., Xu, H., Zhao, M., Zhang, X. L. & Ye, J. H. Co-ZIF-9/TiO_2 nanostructure for superior CO_2 photoreduction activity. *J. Mater. Chem. A* **4**, 15126-15133 (2016).
14. Wan, S. P., Ou, M., Zhong, Q. & Wang, X. M. Perovskite-type $CsPbBr_3$ quantum dots/UiO-66(NH_2) nanojunction as efficient visible-light-driven photocatalyst for CO_2 reduction. *Chem. Eng. J.* **358**, 1287-1295 (2019).
15. Lei, K. et al. A metal-free donor-acceptor covalent organic framework photocatalyst for visible-light-driven reduction of CO_2 with H_2O. *ChemSusChem* 13, 1725-1729 (2020).
16. Xiong, X. Y. et al. Photocatalytic CO_2 reduction to CO over Ni single atoms supported on defect-rich zirconia. *Adv. Energy Mater.* **10**, 2002928 (2020).
17. Yu, X. et al. Photocatalytic reduction of CO_2 to CO over quinacridone/$BiVO_4$ nanocomposites. *ChemSusChem* **13**, 5565-5570 (2020).
18. Xia, Y. et al. Highly selective CO_2 capture and its direct photochemical conversion on ordered 2D/1D heterojunctions. *Joule* **3**, 2792-2805 (2019).
19. Li, X. et al. Direct Z-scheme WO_3/graphitic carbon nitride nanocomposites for the photoreduction of CO_2. *ACS Appl. Nano Mater.* **3**, 1298-1306 (2020).
20. Ji, S. F. et al. Rare-earth single erbium atoms for enhanced photocatalytic CO_2 reduction. *Angew. Chem. Int. Ed.* **59**, 10651-10657 (2020).
21. Cheng, L. et al. Dual-single-atom tailoring with bifunctional integration for high-performance CO_2 photoreduction. *Adv. Mater.* **33**, 2105135 (2021).
22. Li, M. L. et al. Core-shell $LaPO_4$/g-C_3N_4 nanowires for highly active and selective CO_2 reduction. *Appl. Catal. B-Environ.* **201**, 629-635 (2017).
23. Hu, K. et al. Synergetic subnano Ni- and Mn-Oxo clusters anchored by chitosan oligomers on 2D g-C_3N_4 boost photocatalytic CO_2 reduction. *Sol. RRL* **5**, 2000472

(2021).
